# Biofilm mitigation in hybrid chemical-biological upcycling of waste polymers

**DOI:** 10.3389/fbioe.2024.1435695

**Published:** 2024-07-22

**Authors:** Hunter Stoddard, Daniel Kulas, Ali Zolghadr, Sulihat Aloba, Laura G. Schaerer, Lindsay Putman, Isabel Valencia, Jeffrey A. Lacey, David R. Shonnard, Stephen M. Techtmann, Rebecca G. Ong

**Affiliations:** ^1^ Department of Chemical Engineering, Michigan Technological University, Houghton, MI, United States; ^2^ Department of Biological Sciences, Michigan Technological University, Houghton, MI, United States; ^3^ Biological Processing Department, Idaho National Laboratory, Idaho Falls, ID, United States

**Keywords:** plastic, upcycling, chemical-biological recycling, biofilms, biodiversity, pyrolysis, depolymerization

## Abstract

**Introduction:** Accumulation of plastic waste in the environment is a serious global issue. To deal with this, there is a need for improved and more efficient methods for plastic waste recycling. One approach is to depolymerize plastic using pyrolysis or chemical deconstruction followed by microbial-upcycling of the monomers into more valuable products. Microbial consortia may be able to increase stability in response to process perturbations and adapt to diverse carbon sources, but may be more likely to form biofilms that foul process equipment, increasing the challenge of harvesting the cell biomass.

**Methods:** To better understand the relationship between bioprocess conditions, biofilm formation, and ecology within the bioreactor, in this study a previously-enriched microbial consortium (LS1_Calumet) was grown on (1) ammonium hydroxide-depolymerized polyethylene terephthalate (PET) monomers and (2) the pyrolysis products of polyethylene (PE) and polypropylene (PP). Bioreactor temperature, pH, agitation speed, and aeration were varied to determine the conditions that led to the highest production of planktonic biomass and minimal formation of biofilm. The community makeup and diversity in the planktonic and biofilm states were evaluated using 16S rRNA gene amplicon sequencing.

**Results:** Results showed that there was very little microbial growth on the liquid product from pyrolysis under all fermentation conditions. When grown on the chemically-deconstructed PET the highest cell density (0.69 g/L) with minimal biofilm formation was produced at 30°C, pH 7, 100 rpm agitation, and 10 sL/hr airflow. Results from 16S rRNAsequencing showed that the planktonic phase had higher observed diversity than the biofilm, and that *Rhodococcus, Paracoccus,* and *Chelatococcus* were the most abundant genera for all process conditions. Biofilm formation by *Rhodococcus* sp. And *Paracoccus* sp. Isolates was typically lower than the full microbial community and varied based on the carbon source.

**Discussion:** Ultimately, the results indicate that biofilm formation within the bioreactor can be significantly reduced by optimizing process conditions and using pure cultures or a less diverse community, while maintaining high biomass productivity. The results of this study provide insight into methods for upcycling plastic waste and how process conditions can be used to control the formation of biofilm in bioreactors.

## 1 Introduction

Single-use plastic packaging poses a serious environmental concern due to limited environmental biodegradation and rapid turnover of product to waste. Single-use plastics are dominated by polyethylene (PE), polyethylene terephthalate (PET), polypropylene (PP), and polystyrene (PS), which account for 40% of total plastic produced globally (Narancic and O’Connor, 2019). Unfortunately, only 18% of plastic products are currently recycled (Chamas et al., 2020). The remainder is either incinerated, landfilled, or accumulate in natural environments (Chamas et al., 2020). This problem is not expected to abate, as over the last 50 years, plastic waste production has increased by 8.4% each year (Geyer et al., 2017), and plastic waste generation is projected to continue to rise (Satlewal et al., 2008; Borrelle et al., 2020).

Recycling of waste plastics is currently limited because the recycling process does not typically add sufficient value to the resulting product (Ignatyev et al., 2014). Mechanical recycling leads to products of lower physical, mechanical, and aesthetic quality than those produced from virgin feed streams (Sohn et al., 2020). Chemical recycling methods such as pyrolysis, compatibilization, and depolymerization are approaches that have been used to generate valuable products from plastic waste (Zhao et al., 2022). Pyrolysis thermally degrades plastics, such as PE and PP, into shorter straight-chain olefins and paraffins at high temperatures (400°C–700°C) in the absence of oxygen (Kulas et al., 2022), generating gas, liquid, and wax products, which can be used as chemical feedstocks or for energy generation (Ignatyev et al., 2014). Pyrolysis efficiency can be increased by selectively dissolving the waste plastic in a solvent, creating a liquid feed that has improved heat transfer and residence time control compared to a solid feed (Ignatyev et al., 2014; Zolghadr et al., 2022).

In contrast to polyolefins, PET can be depolymerized using chemical processes such as hydrolysis, methanolysis, and glycolysis, and produces monomers or dimers for repolymerization or bioconversion (Yang et al., 2021). The monomers that are generated vary depending on the reactants. For example, when using ammonium hydroxide as the solvent, competing hydrolysis and aminolysis reactions produce terephthalic acid, terephthalic acid monoamide, terephthalamide, and ethylene glycol (Schaerer et al., 2022).

In contrast to chemical approaches, biological depolymerization utilizes microorganisms or enzymes to degrade plastic waste (Ballerstedt et al., 2021). A wide range of organisms have been used to degrade PET (Taniguchi et al., 2019; Wei et al., 2019; Janczak et al., 2020; Kang et al., 2020; Edwards et al., 2022) and PE (Satlewal et al., 2008; Kapri et al., 2010; Park and Kim, 2019; Han et al., 2020), however, biodegradation by microbes alone is slow. It can take as long as 1,200 years for some plastic types (Chamas et al., 2020). To increase biodegradation rates, chemical approaches can first be used to rapidly depolymerize the plastics. The smaller molecular weight products can then be more rapidly converted by the microbes into higher-value products, such as bio-polymers, [e.g., polyhydroxyalkanoate (PHA)] (Guzik et al., 2014; Tiso et al., 2021) or single-cell protein (Schaerer et al., 2022). Previous research has shown that microorganisms are able to utilize the products generated from pyrolysis (Guzik et al., 2014; Byrne et al., 2022) and chemical depolymerization of polymers (Tiso et al., 2021; Sullivan et al., 2022; Schaerer et al., 2023a; Schaerer et al., 2023b; Putman et al., 2023). It may also be possible to increase bioprocessing efficiency by using microbial consortia that employ division of labor to deconstruct mixtures of plastic monomers, leading to greater rates of product formation compared to monocultures (Peng et al., 2016).

Any bioprocessing approach that requires harvest of the microbes, such as production of single-cell protein or generation of an intercellular product, would be negatively affected by the formation of biofilms on surfaces inside the bioreactor (Donlan, 2002). Biofilms are an accumulation of microbial cells that attach to a surface within an enclosed matrix (Leonov et al., 2021). Although biofilms can be beneficial in wastewater treatment systems and biofilm reactors (Qureshi et al., 2005), in most cell cultures they are detrimental, fouling tubing and bioprocessing equipment and leading to decreased system performance and economic sustainability (Garrido-Baserba et al., 2016; Leonov et al., 2021). Systems that use consortia rather than isolates may have greater issues with biofilm formation as the biofilm environment allows for the exchange of nutrients between organisms (Donlan, 2002) and biofilms with greater diversity tend to be thicker (Torresi et al., 2016). Biofilm formation follows a cyclical pattern that includes attachment, growth, multi-layer growth, detachment, and reattachment (Lewandowski et al., 2007). It may be possible to reduce the rate of attachment and the amount of biofilm that is produced by adjusting the pH, temperature, and nutrient levels of the media (Donlan, 2002) or by using chemical control strategies such as biosurfactants and enzymes (Zhu et al., 2022). The challenge is to simultaneously reduce the amount of biofilm while maintaining or increasing the overall productivity of the cell culture.

The objective of this study was to determine the effect of bioprocessing conditions on cell growth and biofilm formation on equipment surfaces during plastic monomer conversion to single-cell protein by a microbial consortium. For these experiments, PET was chemically-depolymerized using ammonium hydroxide, and PP and PE were pyrolyzed to generate carbon-based feedstocks for microbial growth. A microbial consortium (LS1_Calumet), previously enriched from farm compost (Schaerer et al., 2023a), was grown on the depolymerized feedstocks in a stirred-tank bioreactor. The distribution of cell biomass between the biofilm and planktonic state was determined for each bioprocessing condition. High-performance liquid chromatography (HPLC) analysis was used to determine monomer consumption of deconstructed PET (CDPET), and 16S rRNA gene amplicon sequencing was used to determine the most abundant genera and evaluate the diversity of the planktonic and biofilm communities. Biofilm production by the LS1_Calumet consortium was compared to isolates representing two of the most abundant genera, *Rhodococcus* sp. TE21C and *Paracoccus* sp. RL32C (both obtained from a related consortium) when grown on the complex substrate (CDPET) and PET monomers.

## 2 Materials and methods

### 2.1 Pyrolysis of PP and high-density polyethylene

High-density polyethylene (HDPE) (1 cm^3^) and PP (1 cm^3^) were provided by Idaho National Laboratory. A unique liquid feed pyrolysis reactor (Kulas et al., 2022) was designed to have controllable and rapid heat transfer and tunable vapor residence time within the reactor. Heat for the dissolution tank and pyrolysis reactor was provided with XtremeFLEX Heating Tape from BriskHeat (BriskHeat Corporation, Columbus, OH, United States). An Omega Multi-Zone Controller (OMEGA Engineering, Norwalk, CT, United States) was used to measure and control the temperature throughout the length of the stainless-steel tubular (1/4 in. Inside diameter) reactor. The liquid feed was achieved by melting and mixing HDPE and PP (1:1 mass basis) with a pyrolysis wax solvent in the dissolution tank at a 1:1 ratio. Nitrogen gas (99.9% pure) was used for both pressurizing the tank (10 psig) and for inerting the pyrolysis reactor before the reaction. The flow of feed plastic/solvent solution into the plug-flow pyrolysis reactor was pressure-driven and controlled at 0.2 kg/h using a calibrated valve. The vapor residence time was calculated to be approximately 2 s, based on the vapor flow rate through the reactor (Kulas et al., 2022). The temperature within the pyrolysis reactor was maintained at 600°C. Pyrolysis products were cooled through a series of two condensers to separate the products into three groups: a heavy, waxy product (>C15; 24.3% yield); a lighter, liquid product (C5-C15; 38.9% yield); and a gaseous product (C1-C4; 36.9% yield). The liquid products, analyzed by gas chromatography-mass spectrometry (GC-MS), were primarily straight-chain alkenes with some alkanes, alkadienes, and aromatics in smaller quantities (Supplementary Figures S1, S2; Supplementary Table S1). The temperature of the first condenser was maintained at 150°C using the heating tape and controller described previously (Kulas et al., 2022), as well as using a cooling coil on the exterior surface of the condenser. The second condenser was cooled with water to a set point of ∼25°C. The second condenser liquid product was used as the feedstock in the bioreactor experiments described in this document. A batch distillation was used to remove heavier components (>C15) from the Condenser two liquid product.

### 2.2 Chemical deconstruction of PET

PET plastic cups were purchased in bulk from Amazon (Dart TP16D). The cups were initially size reduced at Idaho National Laboratory in a rotary shear (Crumbler^®^, Forest Concepts, Auburn, WA) to 30 mm, then further size reduced to 3–5 mm using the Crumbler^®^. To clean the PET and remove contaminants, milled PET cup particles were added to a custom-built dimethyl ether (DME) extraction vessel. A vacuum from the recovery pump was applied to the chamber until it reached less than −10 psig. Liquid DME was added to the reaction chamber to completely submerge the solids and then held at room temperature for 20 min. The liquid DME was vacuum transferred to another chamber. The solid plastic was removed, dried at room temperature, and stored until use. Aqueous ammonium hydroxide (NH_4_OH: 28–30 wt%, Sigma Aldrich) was diluted with deionized water to the desired concentration of 10 wt%. PET particles were loaded in a custom horizontal batch reactor (550 mL) followed by dilute ammonium hydroxide (0.25 g PET/mL 10 wt% NH_4_OH). Heating tape (HTS/Amptek) was used to heat the reactor to 260°C (monitored using a K-type thermocouple) and then held for a 10 min residence time. Following the residence time, the reactor was cooled using compressed air, and the liquid product was removed from the reactor and vacuum filtered through Whatman #42 filter paper (diameter 55 mm, pore size 2.5 μm). The filtered solids were dried at 55°C and used to determine solubilization based on mass difference, for these experiments achieving 90.5% solubilization of the PET. The liquid was then pH-adjusted from 10.4 to 7 using phosphoric acid and used as a source of C, N, and P for bioprocessing experiments.

### 2.3 Microbial consortium enrichment

Enrichment of the LS1_Calumet culture has previously been described by Schaerer et al. (2023b). Briefly, 1 g of compost from a farm in Calumet, MI (coordinates 47.11, −88.553) was inoculated in 100 mL of 10 g/L disodium terephthalate in Bushnell Haas medium (HiMedia, M350, Kelton, PA). Cultures were incubated at room temperature while stirred continuously with Teflon-coated magnetic stir bars at 130 rpm. The cultures were transferred to fresh medium at 10% inoculum every 14 days over 56 days for a total of four transfers. The enriched culture was then maintained at a 1 L volume with 400 mL of spent culture being replenished with fresh medium every 3–7 days.

### 2.4 Plastic bioconversion

The inoculum culture for the bioconversion experiments was prepared as follows: 10 mL of LS1_Calumet culture was added to a 250 mL Erlenmeyer flask containing 90 mL of fresh media (3.27 g/L Bushnell Haas and 10 g/L disodium terephthalate). The culture grew for 24 h before being used as inoculum for bioconversion in the bioreactors. A mixture of 200 mL of Bushnell Haas Broth (3.27 g/L) and 3 mL of CDPET was added to 250 mL Eppendorf DASbox mini bioreactors equipped with Rushton type impellers and assembled with three sensors for measuring pH (Hamilton^®^ EasyFerm^®^ Plus, autoclavable, O.D. 12 mm, L 120 mm), dissolved oxygen (DO) (Hamilton^®^, autoclavable, O.D. 12 mm, L 120 mm), and optical density (OD_600_) (autoclavable, optical path length 10 mm, L 120 mm) from Eppendorf (Eppendorf North America Inc., Enfield, CT, United States). The entire bioreactor assembly was then autoclaved. A two-point pH calibration (pH 4 and 7) and a one-point DO calibration were performed before each run. Process conditions were controlled using the Eppendorf DASgip system (DASware Control 5). Each run used different experimental process conditions with temperature, pH, mixing, and aeration as variables. The control conditions were 40°C, pH 7, 100 rpm agitation speed, and 0 sL/hr airflow. Experimental conditions altered one variable for each run, with alternative conditions such as 30°C, pH 6 and 8, 750 rpm agitation speed, and 10 sL/hr airflow (Supplementary Table S2). For each run, the bioreactors were allowed to reach stable process conditions (temperature, pH, mixing, airflow rate) before inoculation. The pH was controlled using 20 wt% phosphoric acid and 20 wt% NH_4_OH, diluted from 85 wt% phosphoric acid or 28–30 wt% NH_4_OH, respectively (both supplied by Sigma-Aldrich). Aeration was provided from a compressed gas cylinder of Ultra Zero Grade Air (American Welding and Gas, South Range, MI). Each bioreactor was inoculated with the inoculum culture via micropipette to an initial OD_600_ of 0.05 and allowed to grow for 48 h at the desired process conditions (Supplementary Table S2). Throughout each run, 2 mL samples were taken, at 0, 6, 12, 24, 36, and 48 h, via sterile syringe. OD_600_ was determined using a NanoDrop™ UV-Vis spectrophotometer (Thermo Fisher Scientific Inc., Waltham, MA, United States) at 600 nm with cuvettes.

### 2.5 HPLC analysis

HPLC analyses were performed to determine the amount of substrate consumption by the microbial communities over the 48-h fermentation. During the bioreactor experiments, 2 mL samples were taken at 0 and 48 h using a 3 mL sterile syringe and used to determine the concentrations of deconstructed PET monomers in solution: terephthalic acid, terephthalamide, and terephthalic acid monoamide (Schaerer et al., 2023a). These samples were diluted 100x and filtered through a 0.2-micron PES syringe filter. The samples were stored in 1.5 mL amber HPLC vials at 4°C before HPLC analysis.

Standard solutions of terephthalic acid, terephthalic acid monoamide, and terephthalamide were prepared at a concentration of 1 g/L in dimethylformamide (DMF) and diluted using DMF before filtration through a 0.22 µm PTFE filter. HPLC analysis was performed with an Agilent 1,200 liquid chromatography system equipped with a G1311A quaternary pump, G1322A degasser, G1329 autosampler, G1315B DAD detector, and G1316A temperature column controller. The separations were carried out using a Waters µBondapak C18 column (3.9 mm × 300 mm, 10 µm) with a column temperature of 45°C. The mobile phase consisted of 0.2% formic acid-water solution (A) and 0.1% formic acid-acetonitrile solution (B) with a flow rate of 0.4 mL/min and an injection volume of 10 µL for 25 min. Terephthalic acid and terephthalic acid monoamide were analyzed using the diode array detector (DAD) at 300 nm and terephthalamide at 275 nm. Calibration curves were obtained by plotting the peak area of standards against their known concentration and were used to determine the concentration of monomers in experimental samples.

### 2.6 Biofilm analysis and quantification

Differences in the number of cells suspended in the cell culture and the cells attached as the biofilms were determined on a mass basis. At the end of fermentation, all bioreactor processes were halted and while cells were still suspended in solution, 25 mL of the cell culture was quickly transferred to a 50 mL conical centrifuge tube using a 25 mL serological pipette. A 1 mL sample was also taken for 16S rRNA sequencing (described below), and the remaining cell culture was removed using a 25 mL serological pipette and discarded. To dislodge the biofilm from the bioreactor and probe surfaces, 200 mL of distilled water was added to the reactor and agitated at 1,000 rpm for 10 min. The agitation was then halted, and the reactor was examined to determine if there were any remaining cells attached as a biofilm. If the biofilm remained, the agitation was restarted for another 10 min, until the biofilm cells were homogonously mixed into the solution. Once all the biofilm was detached from the walls of the reactor, a 1 mL sample of the suspended biofilm was taken for 16S sequencing and 25 mL of the biofilm water mixture was transferred to a 50 mL conical centrifuge tube. The planktonic cell culture and biofilm tubes were then centrifuged in a swing-bucket rotor at 3,220× g at 4°C for 30 min (Eppendorf 5810R, Eppendorf North America Inc., Enfield CT). The supernatant was then discarded, and the cell pellets were transferred to weighed, aluminum drying pans and dried in an oven at 105°C for 48 h. The dried pans were then weighed to determine the mass of cells suspended in the plantonic cell culture and the mass of cells attached as biofilm in the reactor.

### 2.7 Extraction of DNA, 16S rRNA gene amplicon sequencing and sequence processing

In order to determine differences in the planktonic and biofilm microbial community composition, planktonic and biofilm biomass samples were collected at the end of each experiment for 16S rRNA gene amplicon sequencing. The 1 mL samples of the planktonic cell culture and resuspended biofilm were transferred to 1.5 mL microcentrifuge tubes and centrifuged at 12,127× g, at room temperature for 5 min in a microcentrifuge (Sorvall MC-12). The supernatant was decanted and discarded, and the cell pellets were stored at −80°C until DNA extraction.

DNA extraction was performed using the ZYMO Research Quick-DNA Fungal/Bacterial Miniprep Kit according to manufacturer instructions. The ZYMO Research *Quick-*16S Plus NGS Library Prep Kit (V3-V4) was used to prepare a DNA library from the ultra-pure DNA samples according to manufacturer instructions. The pooled library was sequenced using an Illumina MiSeq with a MiSeq v3 600 Cycle Reagent Kit.

Sequence processing was carried out in R (R Core Team, 2013) using the DADA2 (divisive amplicon denoising algorithm) package (Callahan et al., 2017). First, primer sequences were filtered and trimmed to remove errors in the data. Low-quality reads were removed using a truncation length of 280 for forward reads, and 180 for reverse reads. Amplicon sequence variants (ASVs) were then inferred and forward and reverse reads were paired together. Chimeras were then removed from the ASVs, and taxonomy was assigned using the SILVA v138.1 training set. Sequences were rarefied using the “rarefy even depth” function of the phyloseq package. Mitochondria and chloroplast DNA were filtered out to remove eukaryotic DNA from the data.

### 2.8 Biofilm production

To quantify biofilm production of the microbial consortium, a microplate test was conducted based on the work performed by Abidi et al. (2013). 1:100 dilutions of LS1_Calumet consortium and media were added (400 µL) to the wells of a 48-well microplate. The types of media used were 6.5 v/v% CDPET, 10 g/L terephthalic acid, and 10 g/L ethylene glycol all with 3.27 g/L Bushnell Haas and water. 20 g/L lysogeny broth was used in three wells as a positive control. Three wells of uninoculated media and three inoculated wells containing Bushnell Haas without any carbon source were used as negative controls. The microplate was covered with a breathable Nunc™ non-woven rayon film (Thermo Fisher Scientific Inc., Waltham, MA, United States) and incubated at 30°C for 5 days in a microplate reader (BioTek Synergy HT, Marshall Scientific LLC., Hampton, NH, United States while monitoring the cell density over time. After the incubation period, each well of the microplate was aspirated to remove any free liquid. To quantify the amount of biofilm in each well, the wells were then washed three times with 500 µL of 1X PBS (pH 7.4). The cells were then stained with 250 µL 0.1% Crystal Violet (w/v) solution. After 10 min, the stain was discarded, the wells were washed three times with distilled water and then allowed to air dry in a desiccator. Once dry, 400 µL of 95% ethanol was added to each well and the plate was incubated without shaking at room temperature for 10 min. The contents of each well were then mixed using a 1,000 µL micropipette and 250 µL of the mixture was transferred to wells in a fresh microplate. Absorbance measurements were taken at 630 nm using a microplate reader. To determine the contribution of individual organisms in the consortium, the microplate test was repeated for the pure strains *Rhodococcus* sp. TE21C and *Paracoccus* sp. RL32C. These strains were isolated from microbial communities capable of utilizing the CDPET liquid product, but not from LS1_Calumet. A representative *Chelatoccocus* sp. Strain was not able to be isolated from the consortium and was not included in the microplate test.

### 2.9 Statstical analysis of bioconversion, biofilm production, and diversity metrics

Analysis of variance (one-way ANOVA) tests were performed using Minitab^®^ Statistical Software v22 to determine significant differences between consumption of terephthtalic acid and terephthalic acid monoamide, biomass cell density, and the percentage of biomass in the biofilm state based on the experimental conditions. Post-hoc Dunnett’s tests were then performed to determine any statistical differences in pairwise comparisons to the control conditions. A two-way ANOVA was also performed on the absorbance readings for biofilm production to determine significant differences between cultures and carbon sources, for both main effects and interactions. Tukey’s pairwise comparisons were used to group cultures grown on the different carbon sources based on statistically significant differences in biofilm formation. The *p*-values reported for the *post hoc* Dunnet and Turkey’s pairwise comparisons are adjusted *p*-values.

Diversity metrics and statistical analyses were carried out in R (R Core Team, 2013). Community diversity metrics were analyzed using the Phyloseq package (McMurdie and Holmes, 2013). Alpha diversity was quantified for each sample using the “Observed” and “Shannon” diversity indices. The Observed index accounts for the richness of a sample (unique taxa), whereas the Shannon index also accounts for evenness (the relative proportions of related taxa in the sample). A principal coordinate analysis (PCoA) ordination plot was used to examine differences in community composition (beta diversity) using the UniFrac distance, between planktonic and biofilm communities for the different process conditions. Lastly, the relative abundances of taxa were compared across the different process conditions. The tidyverse package (Wickham et al., 2019) was used for data analysis and generation of figures from the sequencing data. To determine any significant differences between biomass states (planktonic and biofilm) and between experimental conditions, Kruskal-Wallis tests were performed followed by *post hoc* Dunn tests to determine any significant differences between pairwise comparisons of the process conditions (Kruskal and Wallis, 1952; Dunn, 1964). The *p*-values reported for the *post hoc* Dunn comparisons are adjusted *p*-values. A permutational analysis of variance (PERMANOVA) test using the vegan package (Dixon, 2003) was conducted to determine if biomass states or process conditions had any significant differences in community composition.

## 3 Results

### 3.1 Growth on CDPET and pyrolysis products

**FIGURE 1:**
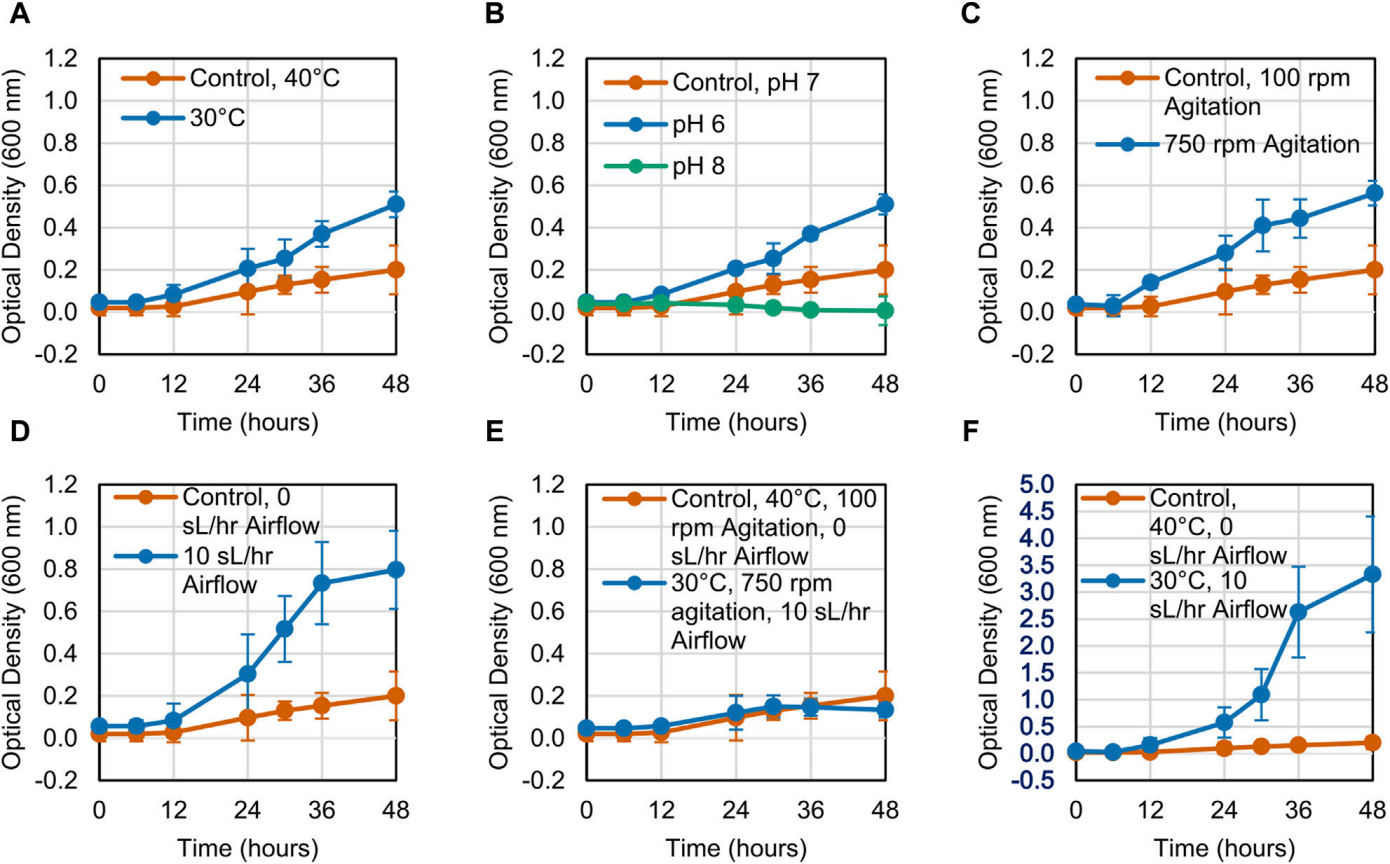
Growth curves for LS1_Calumet cultures grown for 48 h in 200 mL of Bushnell Haas media and 3 mL CDPET under varying conditions of **(A)** temperature, **(B)** pH 6 and pH 8, **(C)** agitation speed, **(D)** airflow rate **(E)** temperature, agitation speed, and airflow, and **(F)** temperature and airflow. The control conditions were 40°C, pH 7, 100 rpm agitation, and 0 sL/hr airflow. Error bars represent average ± standard deviation (n = 3).

**FIGURE 2:**
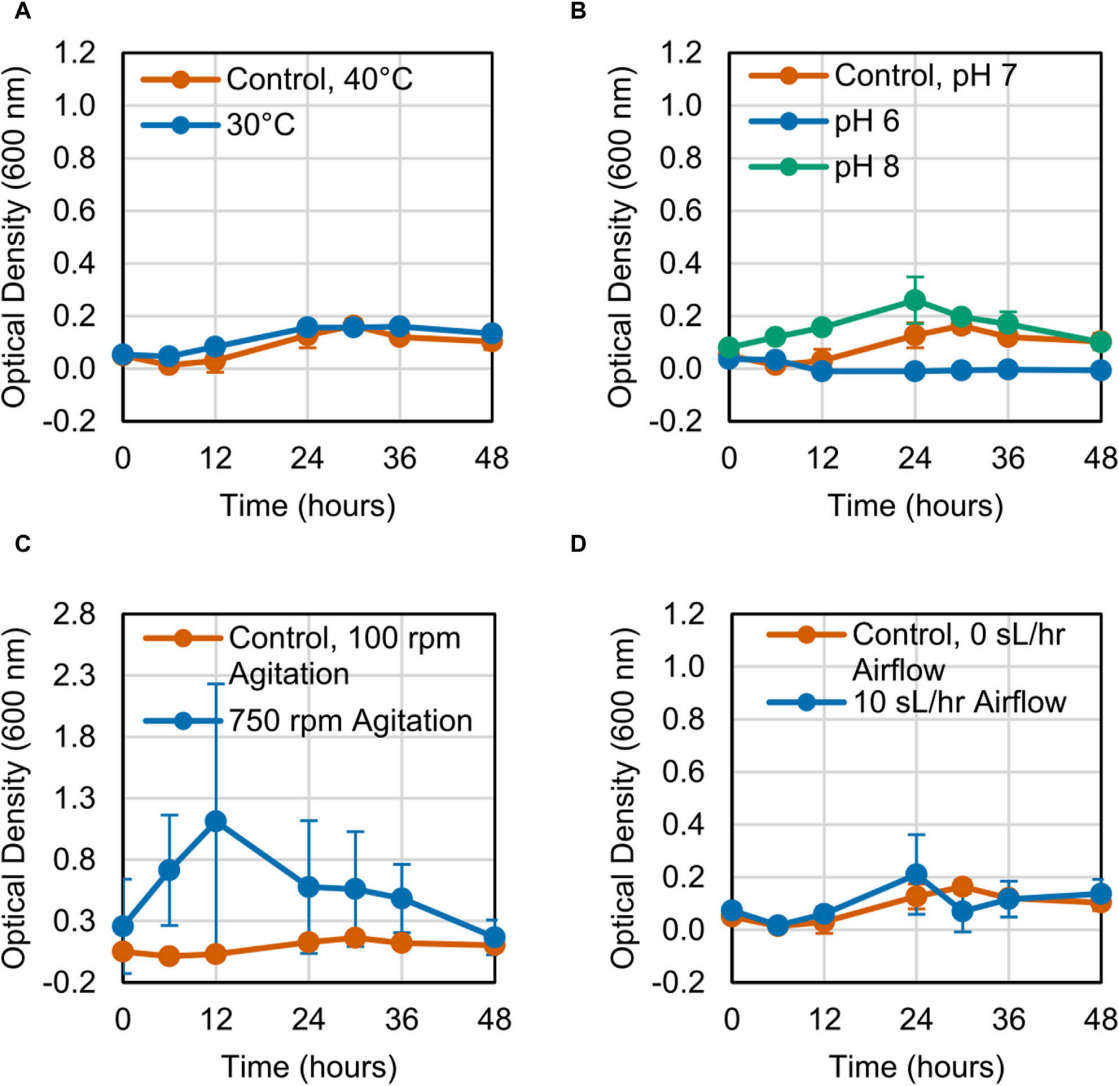
Growth curves for LS1_Calumet cultures grown for 48 h in 200 mL of Bushnell Haas media and 3 mL distilled pyrolysis products under varying conditions of **(A)** temperature, **(B)** pH 6 and pH 8, **(C)** agitation speed, **(D)** and airflow rate. The control conditions were 40°C, pH 7, 100 rpm agitation, and 0 sL/hr airflow. The aerated sample had an airflow rate of 10 sL/hr. Error bars represent average ± standard deviation (n = 3).

The growth of the LS1_Calumet consortium on deconstructed waste plastic substrates was evaluated based on changes in optical density (OD_600_) over 48 h. When altering a single variable, microbial growth on the CDPET was enhanced by lowering the temperature from 40°C to 30°C, increasing agitation from 100 rpm to 750 rpm, and increasing the airflow rate from 0 sL/hr to 10 sL/hr (Figures 1A, C, D). Optical densities were greater at neutral (pH 7) compared to alkaline pH (pH 8) (Figure 1B), however at acidic conditions (pH 6) a white precipitate formed that interfered with optical density readings, leading to high absorbance measurements. When combining the three conditions that showed improved growth (30°C, 750 rpm agitation, and 10 sL/hr airflow), no improvements in growth were observed compared to the control (Figure 1E). In order to determine if the combination of high agitation speed and airflow was exerting too much stress on the cells, the agitation was kept at 100 rpm, while simultaneously reducing the temperature to 30°C and increasing the airflow to 10 sL/hr. This condition had the largest improvement in growth (Figure 1F). In contrast to the CDPET, little to no growth was observed on the pyrolysis liquid product under any of the experimental conditions (Figure 2), likely due to low solubility of the pryrolysis product. Because of the low cell growth on the pyrolysis liquid product, only the cultures grown on the chemically deconstructed PET were evaluated further.

**FIGURE 3:**
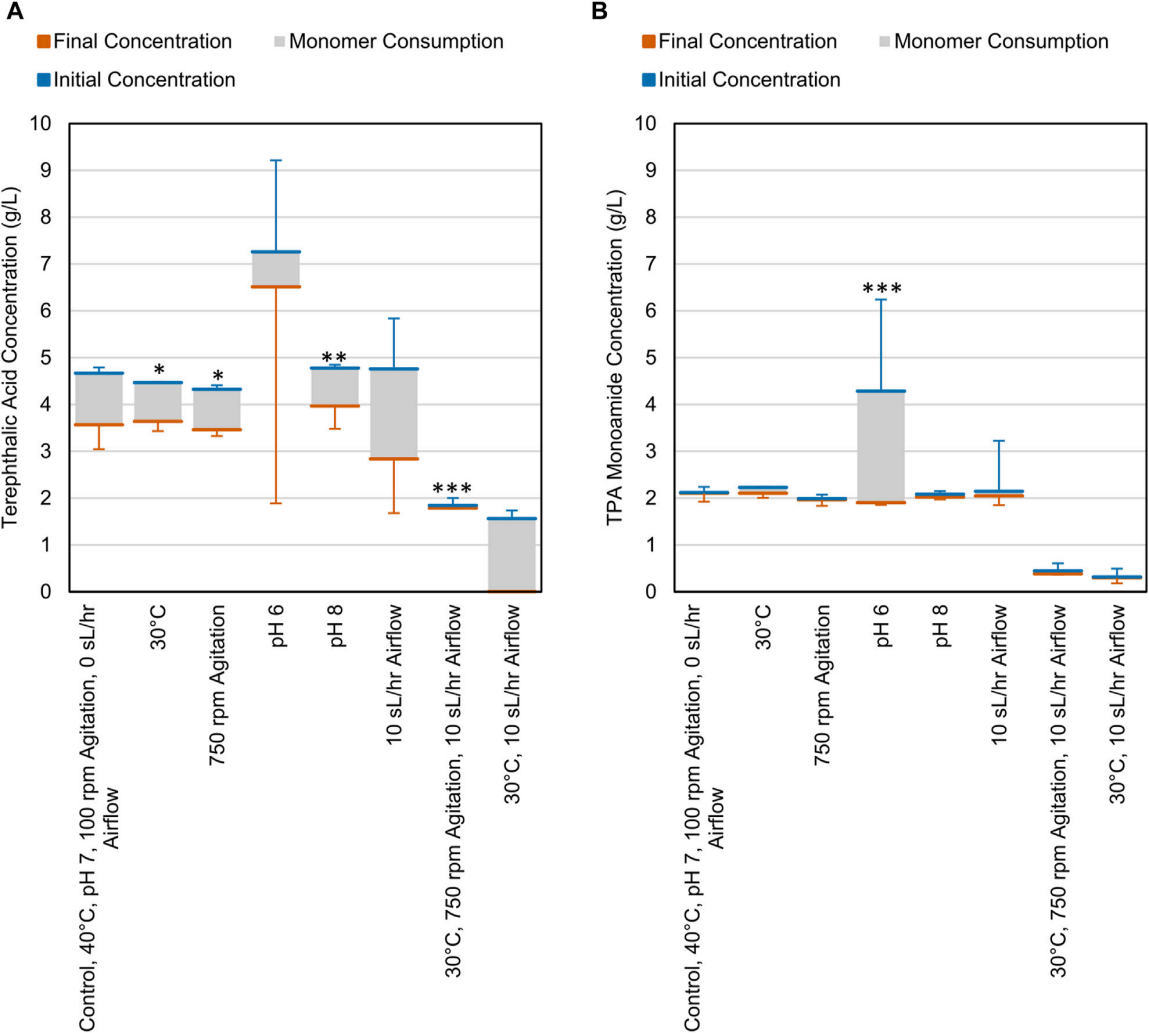
Consumption of **(A)** TPA and **(B)** TPA monoamide during cell cultures grown on CDPET. The blue line represents the initial concentration in the media, the orange line represents the final concentration and the grey area represents the consumption of each monomer. The control conditions were 40°C, pH 7, 100 rpm agitation, and 0 sL/hr airflow. The other conditions were identical to the control except for the parameter mentioned. Error bars represent average ± standard deviation (n = 3). Asterisks denote an experimental condition with statistically different monomer consumption (final–initial) compared to the control (*: *p* < 0.05; **: *p* < 0.01; ***: *p* = 0.000).

CDPET aromatic monomer consumption (terephthalic acid (TPA), terephthalic acid monamide, and terephthalamide) indicated that the consortium was able to utilize ∼1–2 g/L of TPA (Figure 3A) and minimal TPA monoamide (Figure 3B) in the first 48 h. Less TPA was consumed when altering variables to either 30°C, pH 8, 750 rpm, or the combined condition (30°C, 750 rpm, 10 sL/hr airflow) compared to the control (40°C, pH 7, 100 rpm, 0 sL/hr airflow) (Dunnett, *p* < 0.05) (Supplementary Table S3). The remaining conditions showed no significant difference in TPA consumption. For TPA monoamide consumption, only the pH 6 process conditions showed a significant difference compared to the control (an increase; Dunnett, *p* = 0.000) (Supplementary Table S4). As previously stated, white precipitate was observed at pH 6, which is close to the theoretical solubility limit of TPA monoamide (Supplementary Figure S3) (Chemaxon, 2024). Formation of TPA monoamide precipitate would correspond to a decrease in media concentration. No terephthalamide was detected in any of the samples, which aligns with previously reported results that showed no appreciable terephthalamide production from ammonium hydroxide deconstruction of PET (Schaerer et al., 2023b).

### 3.2 Biomass production and distribution between planktonic and biofilm states

**FIGURE 4:**
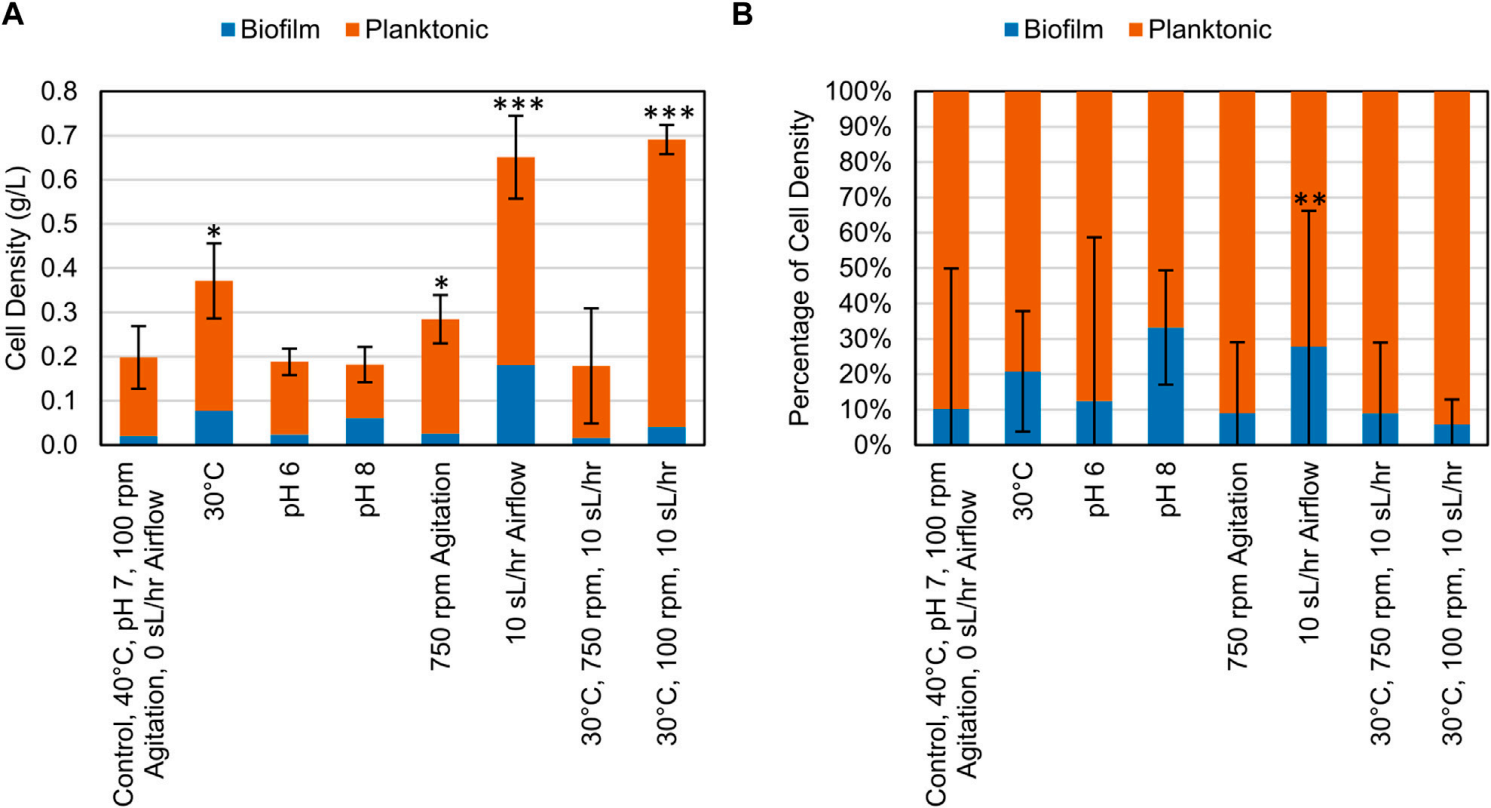
**(A)** Cell density (g/L) of biomass collected at the end of bioconversion of CDPET for various experimental conditions. **(B)** Comparison of the mass of cells suspended in liquid media (planktonic) and as biofilm for various experimental conditions. LS1_Calumet consortium was grown in 200 mL of Bushnell Haas media and 3 mL of CDPET. The control conditions were 40°C, pH 7, 100 rpm agitation, and 0 sL/hr airflow. The other conditions were identical to the control except for the parameter mentioned. Error bars represent average ± standard deviation (n = 3). Asterisks denote an experimental condition with statistical differences compared to the control (*: *p* < 0.05; **: *p* < 0.01; ***: *p* = 0.000).

After the growth period, samples of the planktonic cells and biofilm cells were collected and centrifuged, and the resulting pellets were dried and weighed. When grown on CDPET, most of the biomass was distributed as planktonic cells (Figure 4). A significant increase in total cell density was observed (Dunnett, *p* < 0.05) when altering variables to either 30°C, 750 rpm, 10 sL/hr airflow or the combined change to 30°C and 10 sL/hr airflow compared to the control condition. The percentage of cell mass in the biofilm was only significantly different from the control (Dunnett, *p* = 0.000) for the increased airflow process condition (Supplementary Tables S5, S6).

### 3.3 Taxonomy and microbial community diversity of planktonic and biofilm communities

**FIGURE 5:**
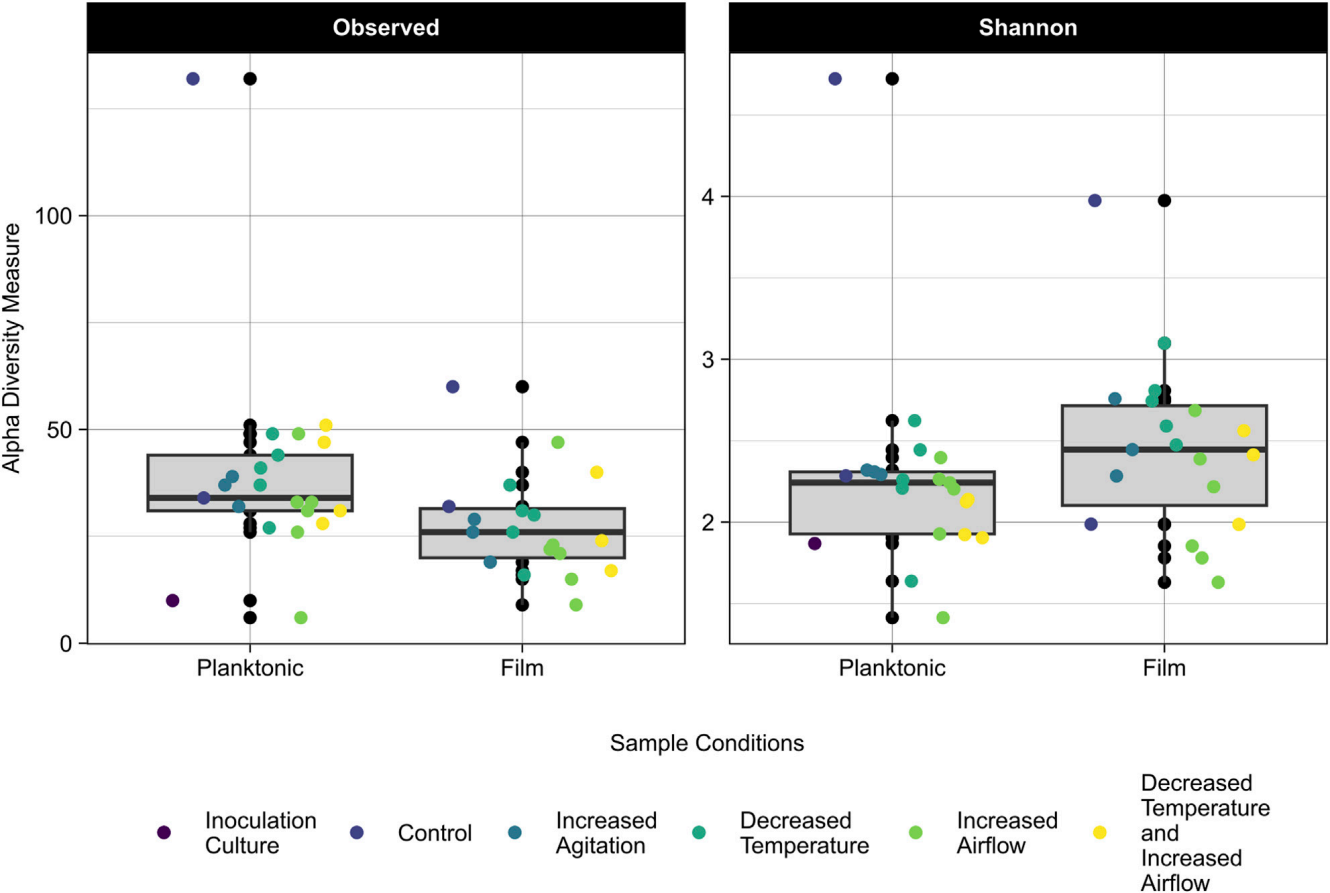
Observed (left) and Shannon Index (right) alpha diversity of the planktonic and biofilm samples for the inoculation culture, control, increased agitation, decreased temperature, increased airflow, and decreased temperature and increased airflow replicates. The grey area represents the interquartile range (IQR) which includes data between 25% and 75% and the median is represented by the black line. Lines extending from the IQR represent the upper and lower limits, with points beyond these limits being outliers in the data.

The 16S rRNA gene amplicon sequencing data was used to assess differences in community composition and taxonomy of samples cultured on CDPET for a subset of experimental treatments: control (40°C, pH 7, 100 rpm, 0 sL/hr airflow) increased agitation (750 rpm), decreased temperature (30°C), increased airflow (10 sL/hr airflow), and the mixed condition of decreased temperature and increased airflow (30°C and 10 sL/hr). The observed alpha diversity (the number of observed taxa in a community) and the Shannon index, which accounts for both the richness and evenness, were determined for the planktonic and biofilm samples (Whittaker, 1972). For samples with the same number of taxa, a smaller Shannon index indicates greater evenness. The observed diversity of the planktonic samples was higher than the biofilm samples (Kruskal-Wallis, *p* = 0.01), but the Shannon indices were not significantly different (Kruskal-Wallis *p* = 0.05) (Figure 5; Supplementary Table S7), indicating that although the number of taxa were higher in the planktonic samples, the proportion of each taxa in the sample (or evenness) was similar for the planktonic and biofilm samples. When comparing the alpha diversity between process conditions, there was no significant difference in observed diversity (Kruskal-Wallis, *p* = 0.12), but a significant difference in the Shannon diversity (Kruskal-Wallis, *p* = 0.04). Pairwise comparisons following *post hoc* Dunn tests showed no significant differences between the process conditions (*p* > 0.05) (Supplementary Table S8).

**FIGURE 6:**
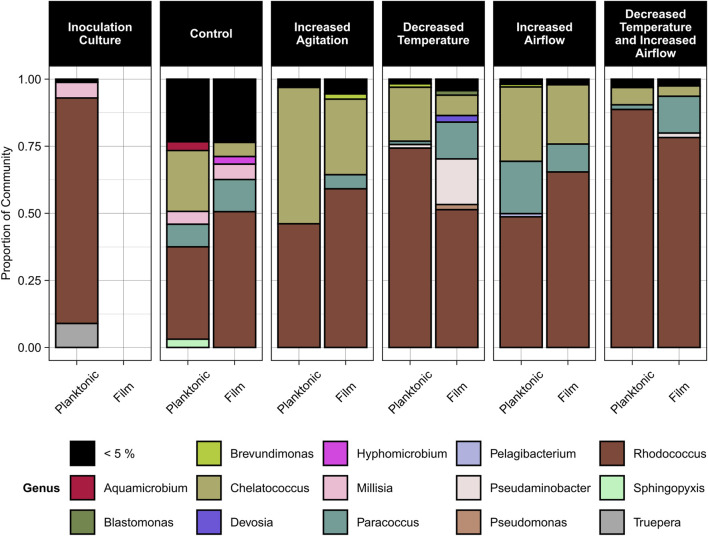
Relative abundance of microbial genera in the planktonic and biofilm states for the inoculation culture, control, increased agitation, decreased temperature, increased airflow, and decreased temperature and increased airflow conditions. The control conditions were 40°C, pH 7, 100 rpm agitation, and 0 sL/hr airflow. The other conditions were identical to the control except for the parameter mentioned.

Based on the sequencing data, three genera dominated the samples, *Rhodococcus* (36%–89% relative abundance), *Chelatococcus* (3%–50% relative abundance), and *Paracoccus* (1%–20% relative abundance) (Figure 6). *Rhodococcus* sp. Increased in relative abundance within the community for any conditions when the temperature decreased to 30°C compared to the control condition of 40°C. Under most conditions except for increased airflow, *Paracoccus* sp. Formed a greater proportion of the community in the biofilm compared to the planktonic state. Under all conditions, *Chelatococcus* sp. Composed a greater proportion of the planktonic community compared to the biofilm community and was more abundant at the higher temperature (40°C) conditions.

A permutational multivariate analysis of variance (PERMANOVA) test was conducted to determine the effects of biofilm state and process conditions on the community composition based on UniFrac distance. There was a significant difference in community composition between planktonic and biofilm samples (*p* = 0.001) as well as between process conditions (*p* = 0.001), with the process conditions having a greater impact on variance than the biomass state (planktonic vs biofilm) (*R* ^2^ of 0.205–0.108 respectively) (Supplementary Table S9). A visualization of the differences in the samples is provided as a principal coordinate analysis (PCoA) plot in Supplementary Figure S4.

### 3.4 Biofilm microtiter-plate test observations

**FIGURE 7:**
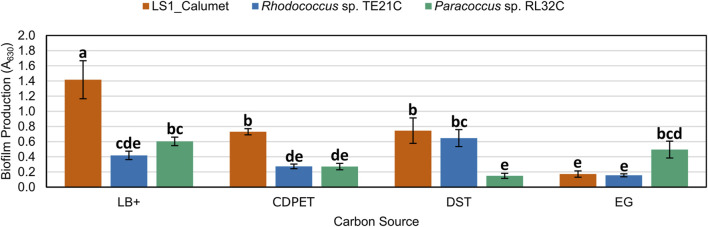
Comparison of biofilm forming potential of LS1_Calumet consortium and *Rhodococcus* sp. TE21C and *Paracoccus* sp. RL32C isolates grown on various carbon sources. Each culture was grown on lysogeny broth as a positive control (LB+), CDPET, and monomeric substrates TPA and ethylene glycol (E.G.) in Bushnell Haas media. A_630_ is the absorbance (630 nm) of crystal violet that was used to stain the biofilm. Error bars represent average ± standard deviation (n = 3). Results are grouped a–e, with results in the same group showing no significant difference between the two based on culture and carbon source.

Following sequencing of the samples, isolates of the most abundant genera, *Rhodococcus* and *Paracoccus*, were obtained from a related CDPET-utilizing consortia, and their biofilm formation (Abidi et al., 2013) was compared to the microbial consortium LS1_Calumet. Although *Chelatococcus* sp. Were also abundant in the cell cultures (Figure 6), we were unable to isolate a representative organism from this genus. Biofilm microplate test results indicate that the LS1_Calumet consortium had the highest biofilm production (A_630_) compared to the isolates (*p* < 0.05) (Figure 7). When compared to CDPET as the sole carbon source, biofilm-forming potential was lower for ethylene glycol (*p* = 0.014), higher for lysogeny broth (*p* = 0.000), and comparable for terephthalic acid (Supplementary Tables S10, S11).

## 4 Discussion

The effect of environmental conditions on biofilm formation has largely been studied as it relates to human health and safety, such as biofilm formation by pathogenic bacteria (Stepanović et al., 2003; Labrie et al., 2010; Jung et al., 2013; Ye et al., 2015) or on food industry equipment (Zhu et al., 2022); or for its importance for solid substrate degradation, such as in wastewater treatment processes (Mahto and Das, 2022). In our study on the production of single-cell protein from waste plastics, biofilms represent a potential reduction in process efficiency and product yields. Changes in process conditions had a significant impact on biofilm formation, largely through shifts in community composition. *Rhodococcus*, *Paracoccus*, and *Chelatococcus* were the most abundant genera present in all samples, and all three genera have previously been implicated in degradation of plastics and associated monomers. The genes for TPA and, E.G., degradation have been identified in *Rhodococcus* sp. DK17 and *R. jostii* RHA1 (Salvador et al., 2019) and *Rhodococcus* sp. Have previously demonstrated the ability to remove terephthalic acid in a wastewater treatment system under aerobic conditions (Ordaz-Cortés et al., 2014). *Paracoccus pantrophus* DSM 2944 and *P. denitrificans* Pd1222 are known to metabolize ethylene glycol (Pal et al., 2024), and *Paracoccus* sp. Were identified as the primary ethylene glycol degraders in a mixed community (Schaerer et al., 2023a). Reports are mixed regarding the ability of *Paracoccus* sp. To degrade aromatic compounds. Pal et al. engineered a TPA degradation pathway into *P. pantrophus* DSM 2944 because it was unable to degrade terephthalic acid (Pal et al., 2024). However, other studies have shown that *Paracoccus* sp. Can utilize bis-(2-hydroxyethyl)-terephthalate as a sole carbon source and degrade PET (Cheng et al., 2022) and polycyclic aromatic hydrocarbons (Liu et al., 2019). *Chelatococcus* sp. E1 isolated from compost was able to degrade polyethylene of various molecular weights (Jeon and Kim, 2013).

The three genera showed distinct preferences for specific process conditions, which affected the overall biomass production. The abundance of *Rhodococcus* sp. Increased when the temperature was reduced to 30°C (Figure 6), which corresponded to an increase in cell biomass (Figure 4), likely indicating *Rhodococcus* sp. Were the key organisms driving biomass production in this system. *Rhodococcus* sp. Typically grow at temperatures ∼30°C and require adaptation to grow above these temperatures (Gilan et al., 2004; de Carvalho, 2012; Suhaila et al., 2013). In contrast, *Chelatococcus* sp. Made up a comparatively larger proportion of the consortium at 40°C. *Chelatococcus* are typically classified as thermophilic (Ibrahim and Steinbüchel, 2010; Yang et al., 2012; Jeon and Kim, 2013), and in one study *Chelatococcus daeguensis* TAD1 growth decreased when the temperature dropped below 45°C (Xu et al., 2014). The *Paracoccus* sp. Abundance appeared unrelated to the process temperature. This aligns with previous studies indicating that these organisms are mesophiles, with similar growth rates at temperatures of 25°C–45°C Figures 1, 3B and a maximum around 35°C (Bachmann et al., 2023).

In contrast to temperature, which had a strong effect on community composition and indirectly affected biofilm production, aeration and agitation had a more direct effect. Increasing either aeration or agitation individually had a positive effect on total biomass production, but a negative effect when increased simultaneously. In contrast, biofilm production tended to increase with increased aeration but decreased with increased agitation. Aeration increases the formation of polysaccharides, which increases the strength of biofilm attachment (Ahimou et al., 2007). When moving bed biofilm reactors were held under continuous aeration, the biofilms that formed were less prone to detachment compared to systems with intermittent aeration (Wang et al., 2021). Previous work has also suggested that an even dissolved oxygen distribution can increase the biofilm density (Xiao et al., 2021). In contrast, agitation above a certain level typically disrupts biofilm formation, reducing the time to fouling in membrane bioreactors (Khan and Visvanathan, 2008). Shear stresses on the biofilm disrupt its formation and stability, decreasing biofilm mass and thickness (Paul et al., 2012; Wang et al., 2021), and beyond a certain critical threshold, breaking bio-flocs into particles of smaller size (Khan and Visvanathan, 2008). However, excessive shear stresses in the media can also limit microbial viability. Similar to our results, increasing agitation speed and airflow simultaneously increase shear stresses on cells to the point of reducing cell growth, viability, substrate utilization, and product formation (Liao et al., 2012; Paul et al., 2012; Hong et al., 2020; Hong et al., 2020)

The microbial consortia was able to consume ∼1–2 g/L TPA (Figure 3A) for all process conditions where the cells were able to grow (Figure 1). In contrast there was little to no utilization of terephthalic acid monoamide (Figure 3B). The apparent consumption of TPA monoamide and increase in OD_600_ at the pH 6 condition (Figures 1, 3B) is likely attributed to the monoamide crashing out of solution and resulting in the white precipitate that was observed during these experiments. A previous study suggested that phthalate degradation pathways may be used during terephthalamide degradation by *Rhodococcus* sp. (Schaerer et al., 2023a). However, the pathways for TPA monoamide and terephthalamide degradation are not well characterized within the current literature. It is possible the required pathway is not present within our consortium. The organisms may be missing the enzyme required to convert the amide into a carboxylic acid or may not have the transporters needed to take up the amide from the media. Alternatively, the microbes may preferentially utilize the acid over the monoamide. In one study, compounds at higher concentrations were utilized first (Wang et al., 2022), which if true in our system suggests that terephthalic acid metabolism would occur before the monoamide.

The results in Figure 5 show that the planktonic community has a higher richness and lower evenness than the biofilm communities. In a study comparing the biodiversity between planktonic and biofilm communities, it was found that there was a higher species richness in the planktonic communities than the biofilm communities (Parfenova et al., 2013), which supports the findings of our study. It has also been found that biofilms of greater thickness have a more evenly distributed community (Torresi et al., 2016). Our results showed that the more diverse community of organisms formed more biofilm than the pure strains (Figure 7). Biofilms with multiple species have the benefit of being able to cycle various nutrients (nitrogen, sulfur, and carbon) due to the greater range of metabolic capabilities that a diverse community would have compared to a pure culture (Donlan, 2002). A biofilm community of higher diversity was more resilient to environmental disturbances (changes in pH) than less diverse communities (Feng et al., 2017), suggesting that the community resilience may also be correlated to alpha diversity. Of the genera identified, *Paracoccus* sp. Showed a higher abundance in the biofilm samples compared to the planktonic samples and *Paracoccus* sp. Have previously shown growth in high abundance in biofilm communities (Singh et al., 2015). *Rhodococcus* sp. Can also adhere to solid substrates but strength of adhesion varies depending on the species (Ivshina et al., 2022).

Possible applications of the results in this study are in controlling biofilm formation in different reactor types. For suspended cell systems such as a CSTR, mitigation of biofilm formation can be controlled by changing process conditions to be less favorable for biofilm formation, such as lower airflow rates. In systems where the presence of biofilm is favorable such as biofilter and membrane biofilm reactors, using a microbial community with high diversity can lead to better stability. Our results also suggest that in aerobic processes that require significant oxygenation to maintain cell growth, simultaneously increasing airflow and agitation may negatively impact cell growth and viability, and agitation may need to be reduced to maintain cell viability.

## 5 Conclusion

This study determined that process conditions impact substrate utilization and biofilm formation of a microbial community in a hybrid chemical-biological upcycling system. Temperature and airflow played a key role in the formation of biofilm, with temperature effects largely driven by changes in community composition. Of all the factors, increasing airflow resulted in the largest increases in total cell density. It was also determined that the diverse community of LS1_Calumet was more prone to forming biofilms than the pure cultures of *Rhodococcus* sp. TE21C and *Paracoccus* sp. RL32C. These findings suggest that for this process, either using lower temperatures with higher airflow rates or using pure cultures or a less diverse community, may reduce the formation of biofilm while maintaining high biomass productivity. Future studies using microbial communities of varying diversities, or communities enriched from other sources would further explain the role of diversity in biofilm formation.

## Data Availability

The data presented in the study are deposited in the European Bioinformatics Insistute repositroy (https://www.ebi.ac.uk/), accession number PRJEB77013.
