# Supplementary material for: Biofilm mitigation in hybrid chemical-biological upcycling of waste polymers
Source: Front Bioeng Biotechnol. 2024 Jul 22;12:1435695. doi: 10.3389/fbioe.2024.1435695 (PMC11298394; doi:10.3389/fbioe.2024.1435695)
Supplement: Supplementary file 1 [file DataSheet1.docx]

Supplementary Material

# Supplementary Figures


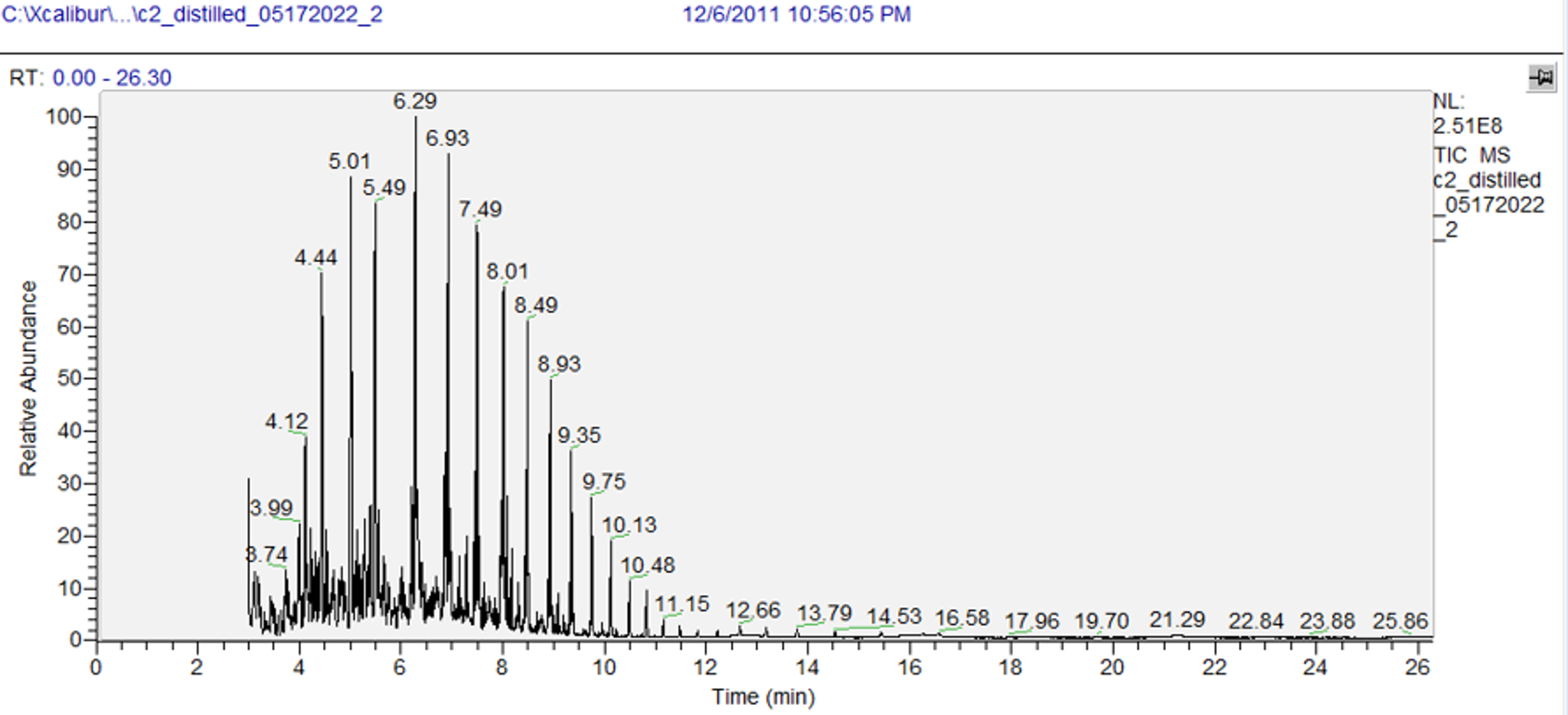


**Figure S1.** GC-MS chromatogram for pyrolysis liquid samples used in biofilm mediation experiments. Batch 1 05/17/2022.


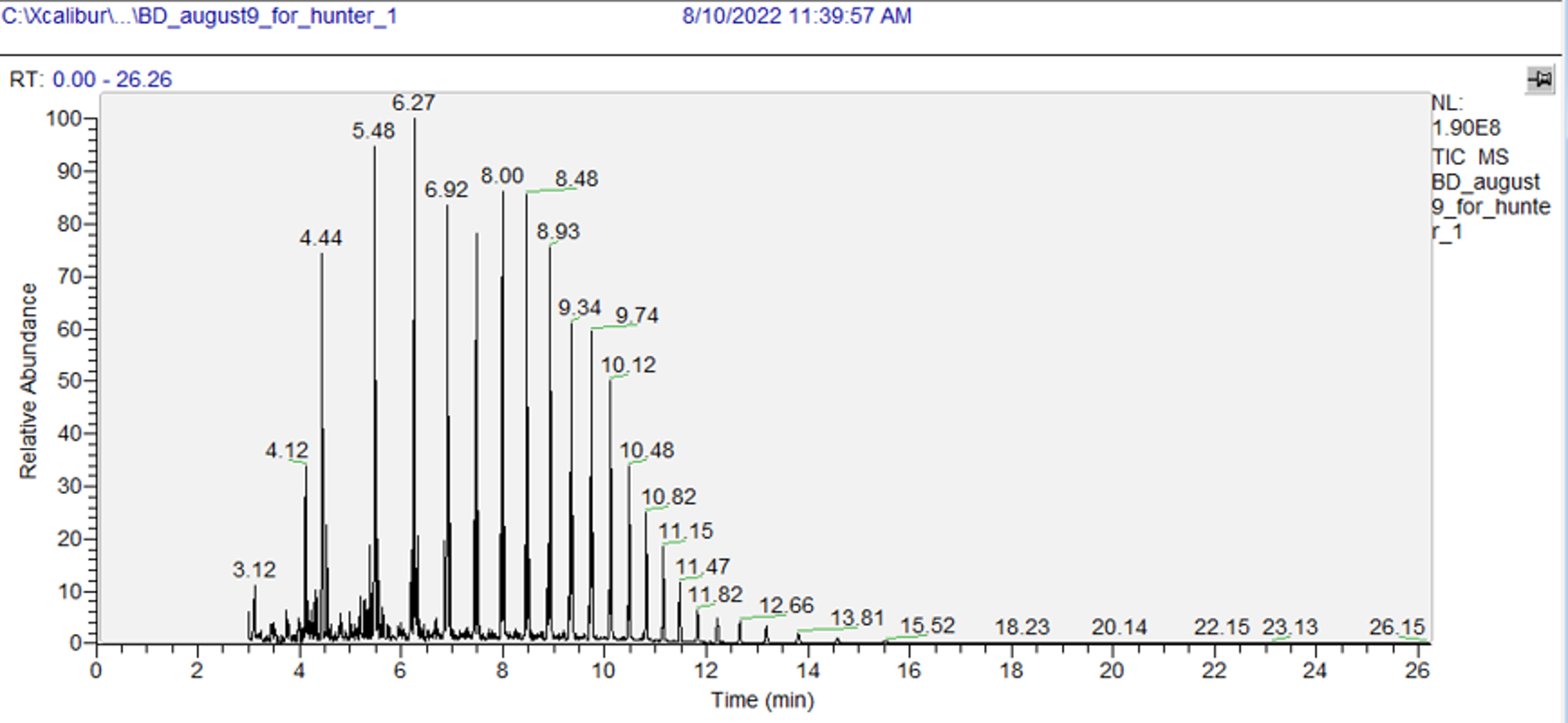


**Figure S2.** GC-MS chromatogram for pyrolysis liquid sample used in biofilm mediation experiments. Batch 08/09/2022.


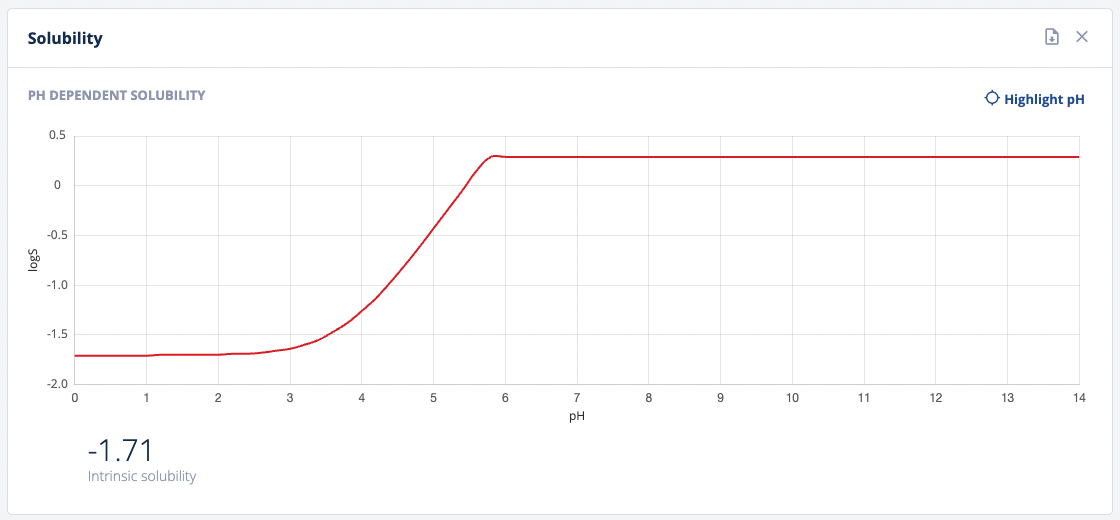


**Figure S3.** Terephthalic acid monoamide solubility. Figure generated using ChemAxon Protonation and Solubility Calculators.


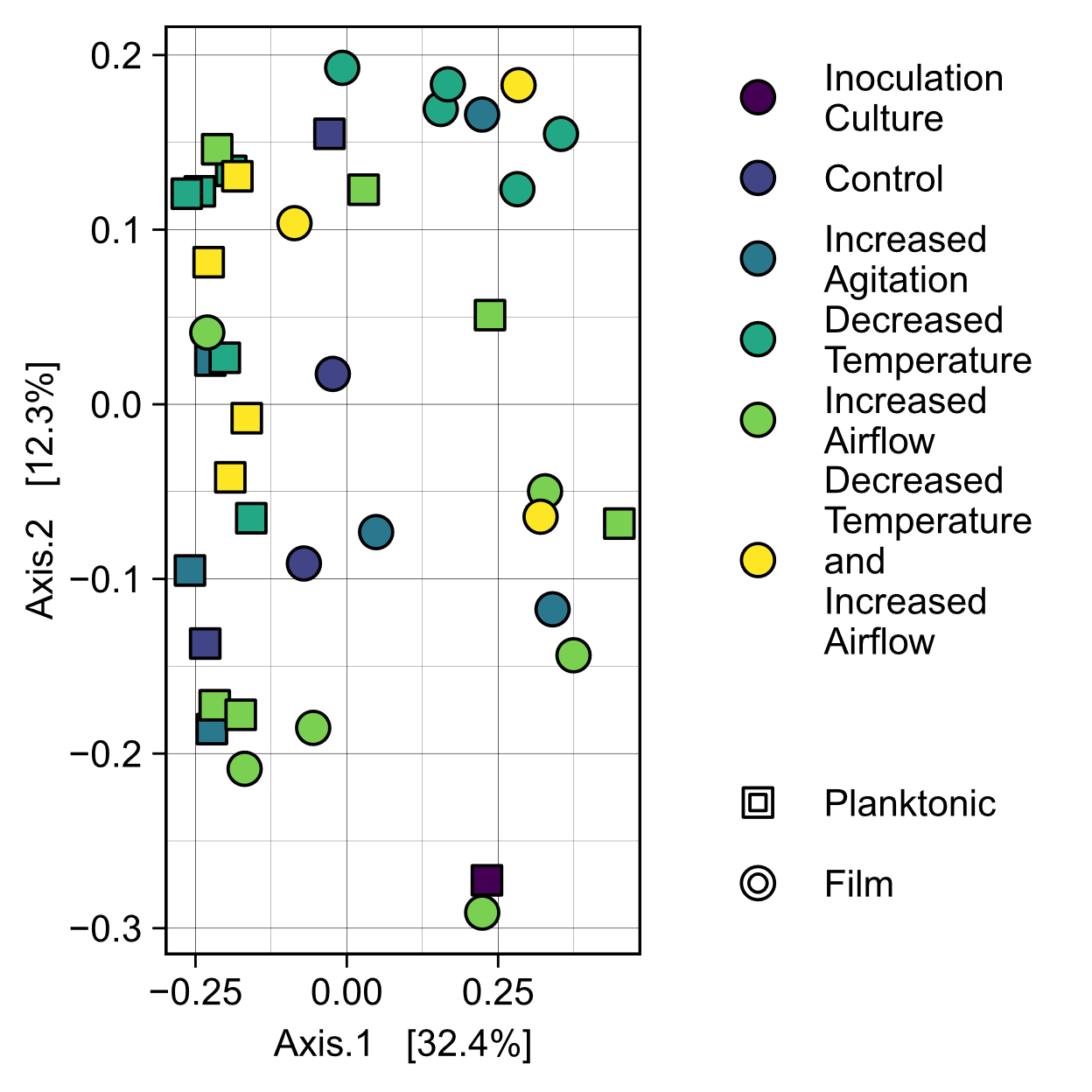


**Figure S4.** Principle coordinates analysis plot demonstrating the differences between planktonic and biofilm samples based on UNIFRAC distance for cultures grown under different operating conditions.

# Supplementary Tables

**Table S1.** Key identified GC-MS peaks in pyrolysis oil

| **Apex Response Time (min)** | **Chemical Name** | **Apex Response Time (min)** | **Chemical Name** |
| --- | --- | --- | --- |
| 4.44 | 1-Octene | 8.93 | 1-Pentadecene |
| 5.48 | 1-Nonene | 9.34 | 1-Hexadecene |
| 6.28 | 1-Decene | 9.74 | 1-Heptadecene |
| 6.92 | 1-Undecene | 10.13 | 1-Octadecene |
| 7.49 | 1-Dodecene | 10.48 | 1-Nonadecene |
| 8.00 | 1-Tridecene | 10.83 | 1-Eicosene |
| 8.49 | 1-Tetradecene | 11.15 | 1-Heneiscosene |

**Table S2.** Bioreactor Operating Conditions for Plastic Bioconversion.

| **Experiment** | **Temperature (°C)** | **pH** | **Agitation Speed (rpm)** | **Airflow (sL/hr)** |
| --- | --- | --- | --- | --- |
| Control | 40 | 7 | 100 | 0 |
| Decreased Temperature | 30 | 7 | 100 | 0 |
| pH 6 | 40 | 6 | 100 | 0 |
| pH 8 | 40 | 8 | 100 | 0 |
| Increased Agitation Speed | 40 | 7 | 750 | 0 |
| Increased Airflow | 40 | 7 | 100 | 10 |
| Decreased Temperature, Increased Agitation Speed, and Increased Airflow | 30 | 7 | 750 | 10 |
| Decreased Temperature and Increased Airflow | 30 | 7 | 100 | 10 |

**Table S3.** Statistical significance of consumption of terephthalic acid between experimental conditions compared to the control using the Dunnett test for pairwise comparisons to the control conditions following analysis of variance (ANOVA) test for significant difference. Asterisks denote an experimental condition with statistically different monomer consumption (final – initial) compared to the control (*: p < 0.05; **: p < 0.01; ***: p = 0.000; NS = not significant).

| **Variable** | **Degrees of Freedom** | **Sum of  Squares** | | **Mean  Squares** | **F Score** | | **p-value** | |
| --- | --- | --- | --- | --- | --- | --- | --- | --- |
| Experimental Conditions | 7 | 10.477 | | 1.497 | 14.77 | | **0.000***** | |
| Residuals | 16 | 1.621 | | 0.101 |  | |  | |
| Total | 23 | 12.098 | |  |  | |  | |
| **Pairwise Comparison vs. Control** | | | **T Statistic** | | | **Adjusted p-value** | |  |
| Decreased Temperature | | | -3.02 | | | **0.042** | | ***** |
| pH 6 | | | 1.56 | | | 0.493 | | NS |
| pH 8 | | | -5.20 | | | **0.001** | | ****** |
| Increased Agitation Speed | | | -3.21 | | | **0.029** | | ***** |
| Increased Airflow | | | -0.14 | | | 1.000 | | NS |
| Decreased Temperature, Increased Agitation Speed, and Increased Airflow | | | -6.08 | | | **0.000** | | ******* |
| Decreased Temperature and Increased Airflow | | | -0.50 | | | 0.994 | | NS |

**Table S4.** Statistical significance of consumption of TPA monoamide between experimental conditions compared to the control using the Dunnett test for pairwise comparisons to the control conditions following analysis of variance (ANOVA) test for significant difference. Asterisks denote an experimental condition with statistically different monomer consumption (final – initial) compared to the control (*: p < 0.05; **: p < 0.01; ***: p = 0.000; NS = not significant).

| **Variable** | **Degrees of Freedom** | **Sum of  Squares** | | **Mean  Squares** | **F Score** | | **p-value** | |
| --- | --- | --- | --- | --- | --- | --- | --- | --- |
| Experimental Conditions | 7 | 2.887 | | 0.412 | 13.26 | | **0.000***** | |
| Residuals | 16 | 0.498 | | 0.031 |  | |  | |
| Total | 23 | 3.385 | |  |  | |  | |
| **Pairwise Comparison vs. Control** | | | **T Statistic** | | | **Adjusted p-value** | |  |
| Decreased Temperature | | | -0.50 | | | 0.995 | | NS |
| pH 6 | | | 6.68 | | | **0.000** | | ******* |
| pH 8 | | | -1.05 | | | 0.826 | | NS |
| Increased Agitation Speed | | | -0.74 | | | 0.957 | | NS |
| Increased Airflow | | | 1.28 | | | 0.674 | | NS |
| Decreased Temperature, Increased Agitation Speed, and Increased Airflow | | | -0.70 | | | 0.967 | | NS |
| Decreased Temperature and Increased Airflow | | | -0.52 | | | 0.993 | | NS |

**Table S5.** Statistical significance of biomass cell density for experimental conditions compared to the control using the Dunnett test for pairwise comparisons to the control conditions following analysis of variance (ANOVA) test for significant difference. Asterisks denote an experimental condition with statistically different total cell density compared to the control (*: p < 0.05; **: p < 0.01; ***: p = 0.000; NS = not significant).

| **Variable** | **Degrees of Freedom** | **Sum of  Squares** | | **Mean  Squares** | | **F Score** | **p-value** | |
| --- | --- | --- | --- | --- | --- | --- | --- | --- |
| Experimental Conditions | 7 | 1.841 | | 0.263 | | 62.30 | **0.000***** | |
| Residuals | 16 | 0.068 | | 0.004 | |  |  | |
| Total | 23 | 1.909 | |  | |  |  | |
| **Pairwise Comparison** | | | **T Statistic** | | **Adjusted p-value** | | |  |
| Decreased Temperature – Control | | | 3.26 | | **0.026** | | | ***** |
| pH 6 – Control | | | -0.19 | | 1.000 | | | NS |
| pH 8 – Control | | | 1.63 | | 0.448 | | | NS |
| Increased Agitation Speed – Control | | | 3.09 | | **0.036** | | | ***** |
| Increased Airflow – Control | | | 8.54 | | **0.000** | | | ******* |
| Decreased Temperature, Increased Agitation Speed, and Increased Airflow – Control | | | -0.36 | | 0.999 | | | NS |
| Decreased Temperature and Increased Airflow – Control | | | 15.74 | | **0.000** | | | ******* |

**Table S6.** Statistical significance of the proportion of cell density in biofilm for experimental conditions compared to the control using the Dunnett test for pairwise comparisons to the control conditions following analysis of variance (ANOVA) test for significant difference. Asterisks denote an experimental condition with statistically different proportion of cells in biofilm compared to the control (*: p < 0.05; **: p < 0.01; ***: p = 0.000; NS = not significant).

| **Variable** | **Degrees of Freedom** | **Sum of  Squares** | | **Mean  Squares** | | **F Score** | **p-value** | |
| --- | --- | --- | --- | --- | --- | --- | --- | --- |
| Experimental Conditions | 7 | 0.122 | | 0.017 | | 4.30 | **0.007**** | |
| Residuals | 16 | 0.064 | | 0.004 | |  |  | |
| Total | 23 | 0.187 | |  | |  |  | |
| **Pairwise Comparison** | | | **T Statistic** | | **Adjusted p-value** | | |  |
| Decreased Temperature – Control | | | 2.54 | | 0.104 | | | NS |
| pH 6 – Control | | | 0.74 | | 0.959 | | | NS |
| pH 8 – Control | | | 1.29 | | 0.673 | | | NS |
| Increased Agitation Speed – Control | | | 0.18 | | 1.000 | | | NS |
| Increased Airflow – Control | | | 3.79 | | **0.009** | | | ****** |
| Decreased Temperature, Increased Agitation Speed, and Increased Airflow – Control | | | 0.12 | | 1.000 | | | NS |
| Decreased Temperature and Increased Airflow – Control | | | -0.52 | | 0.993 | | | NS |

**Table S7**. Statistical significance of the difference in alpha diversity metrics (Observed and Shannon) between culture types (planktonic vs. biofilm) based on Kuskall Wallis rank sum test. Asterisks denote an experimental condition with statistically different proportion of cells in biofilm compared to the control (*: p < 0.05; **: p < 0.01; ***: p = 0.000; None = not significant).

| **Diversity Metric** | **χ^2^** | **Degrees of freedom** | **p-value** |
| --- | --- | --- | --- |
| Observed Diversity | 6.1506 | 1 | **0.01*** |
| Shannon Diversity | 3.75 | 1 | 0.05 |

**Table S8.** Statistical significance of the difference in alpha diversity metrics (Observed and Shannon) between culture conditions. Dunn tests were carried out for significant Kruskal-Wallis comparisons with Benjamini-Hochberg correction. Asterisks denote an experimental condition with statistically different proportion of cells in biofilm compared to the control (*: p < 0.05; **: p < 0.01; ***: p = 0.000; None = not significant).

| **Diversity Metric** | | **χ^2^** | | **Degrees of freedom** | | **p-value** | |
| --- | --- | --- | --- | --- | --- | --- | --- |
| Observed Diversity | | 8.781 | | 5 | | 0.12 | |
| Shannon Diversity | | 11.902 | | 5 | | **0.04*** | |
| **Z-test statistic (p-value)** | **Decreased Temperature** | **Increased Agitation** | **Increased Airflow** | | **Decreased Temp & Increased Airflow** | | **Inoculum Culture** |
| Control | 0.123 (0.902) | 0.276 (0.978) | 1.988 (0.234) | | 1.682 (0.232) | | 1.664 (0.206) |
| Decreased Temperature | -- | 0.204 (0.967) | 2.511 (0.181) | | 1.991 (0.348) | | 1.705 (0.265) |
| Increased Agitation | -- | -- | 1.934 (0.197) | | -1.574 (0.216) | | 1.557 (0.199) |
| Increased Airflow | -- | -- | -- | | 0.197 (0.904) | | 0.685 (0.673) |
| Decreased Temp & Increased Airflow | -- | -- | -- | | -- | | 0.754 (0.676) |

**Table S9.** Permutational multivariate analysis of variance (PERMANOVA) results comparing the differences in community composition between sample types (planktonic vs. biofilm) and process conditions using the unweighted UniFrac method. Asterisks denote if there is a statistical difference between sample types or between operating conditions (*: p < ).05; **: p < 0.01; ***: p =0.000; None = not significant).

| **Variable** | **Degrees of Freedom** | **Sum of Squares** | **Mean Squares** | **F Score** | **R^2^** | **p-value** |
| --- | --- | --- | --- | --- | --- | --- |
| Sample Type | 1.000 | 0.679 | 0.679 | 5.170 | 0.108 | **0.001**** |
| Process Conditions | 5.000 | 1.293 | 0.259 | 1.970 | 0.205 | **0.001**** |
| Residuals | 33.000 | 4.333 | 0.131 |  | 0.687 |  |
| Total | 39.000 | 6.305 |  |  | 1.000 |  |

**Table S10.**  Analysis of variance (ANOVA) test for significant differences between absorbance readings (A_630_) measuring biofilm production of different cultures and carbon sources. Asterisks denote an experimental condition with statistically different proportion of cells in biofilm compared to the control (*: p < 0.05; **: p < 0.01; ***: p = 0.000; NS = not significant).

| **Variable** | **Degrees of Freedom** | **Sum of  Squares** | **Mean  Squares** | **F Score** | **p-value** |
| --- | --- | --- | --- | --- | --- |
| Culture | 2 | 1.213 | 0.606 | 56.12 | **0.000***** |
| Carbon Source | 3 | 1.389 | 0.463 | 42.85 | **0.000***** |
| Culture*Carbon Source | 6 | 1.731 | 0.288 | 26.70 | **0.000***** |
| Residuals | 24 | 0.259 | 0.011 |  |  |
| Total | 35 | 4.593 |  |  |  |

Table S11. Statistical significance of the proportion of cell density in biofilm for experimental conditions compared to the control using Turkey’s pairwise comparisons following analysis of variance (ANOVA) test for significant difference between main effects of cultures and carbon sources. Asterisks denote an experimental condition with statistically different proportion of cells in biofilm compared to the control (*: p < 0.05; **: p < 0.01; ***: p = 0.000; NS = not significant).

|  | **Pairwise Comparison** | **T Statistic** | **Adjusted p-value** |  |
| --- | --- | --- | --- | --- |
| Culture | *Paracoccus* sp. RL32C – LS1_Calumet | -9.11 | **0.000** | *** |
|  | *Rhodococcus* sp. TE21C – LS1_Calumet | -9.24 | **0.000** | *** |
|  | *Rhodococcus* sp. TE21C – *Paracoccus* sp. RL32C | -0.14 | 0.990 | NS |
| Carbon Source | Terephthalic Acid – CDPET | 1.81 | 0.295 | NS |
|  | Ethylene Glycol – CDPET | -3.07 | **0.025** | * |
|  | Lysogeny Broth – CDPET | 7.91 | **0.000** | *** |
|  | Ethylene Glycol – Terephthalic Acid | -4.88 | **0.000** | *** |
|  | Lysogeny Broth – Terephthalic Acid | 6.11 | **0.000** | *** |
|  | Lysogeny Broth – Ethylene Glycol | 10.98 | **0.000** | *** |
